# Public Perceptions of Flavored Waterpipe Smoking on Twitter

**DOI:** 10.3390/ijerph20075264

**Published:** 2023-03-27

**Authors:** Juan Ramon Feliciano, Dongmei Li, Zidian Xie

**Affiliations:** 1 Department of Computer Science, University of Rochester, Rochester, NY 14620, USA; 2 Department of Clinical and Translational Research, University of Rochester Medical Center, Rochester, NY 14642, USA

**Keywords:** waterpipe, flavor, Twitter, smoking

## Abstract

Waterpipe tobacco smoking has become increasingly popular in recent years, especially among youth. We aimed to understand longitudinal trends in the prevalence and user perception of waterpipes and their flavors on Twitter. We extracted waterpipe-related tweets from March 2021 to May 2022 using the Twitter Streaming API and classified them into promotional tweets and non-promotional tweets. We examined the longitudinal trends regarding the waterpipe flavors mentioned on Twitter and conducted sentiment analysis on each waterpipe flavor-related non-promotional tweet. Among over 1.3 million waterpipe-related tweets, 1,158,884 tweets were classified as non-promotional and 235,132 were classified as promotional. The most frequently mentioned waterpipe flavor groups were fruit (34%), sweets (17%), and beverages (15%) among all flavor-containing non-promotional tweets (17,746 tweets). The least mentioned flavor groups were tobacco (unflavored, 4%) and spices (2%). Sentiment analysis showed that among non-promotional waterpipe-related tweets, 39% were neutral, 36% were positive, and 23% were negative. The most preferred waterpipe flavors were fruit, mixed, and alcohol flavors. The least preferred flavor groups were tobacco and spice flavors. Our study provided valuable information on the prevalence of waterpipe flavors that can be used to support the future regulation of flavored waterpipe tobacco products given the nature of the current regulations on other flavored tobacco products.

## 1. Introduction

Tobacco smoking is a global epidemic that leads to the loss of roughly 8 million lives per year globally and 480,000 lives per year in the United States from various cancers, cardiovascular diseases, and heart diseases [1]. Recent studies have shown that compared to non-tobacco smokers, tobacco smokers have significantly increased risks of cardiovascular disease, second primary lung cancer, and chronic musculoskeletal pain [2,3,4]. Furthermore, in addition to established findings on increased risks of all-cause mortality, recent studies have shown that tobacco use is associated with higher rates of COVID-19 mortality [4,5]. The most popular medium of tobacco smoking in 2020 in the United States was cigarettes, followed by e-cigarettes, cigars, smokeless tobacco products, and waterpipes [6]. Although cigarette smoking remains the most common form of tobacco smoking in the United States, it has become less prevalent among the youth in recent years, largely due to the availability of attractive flavors for other tobacco products [7].

Originating in the Middle East, waterpipe tobacco smoking (WTS), known colloquially as hookah, shisha, nargile, and argileh smoking, is a method of tobacco intake in which users inhale coal-generated tobacco smoke that has passed through water. In the United States, waterpipes are an increasingly relevant tobacco product among young adults and have displayed similar current-use patterns to cigarettes among middle-school and high-school students as of 2021 [7]. Waterpipe use in the United States has displayed an upward trend from 2011 to 2019 and has remained unchanged or increased in 19 of 38 nations that collected surveys on waterpipe use between 2000 and 2019 [8]. In 2021, more than 180,000 high schoolers and middle schoolers reported using the product in the past 30 days [7], and the number of waterpipe lounges located around college campuses has risen more than 400% since 1999 [9]. Therefore, waterpipe tobacco smoking has become a global epidemic, especially among youth [10,11].

In addition to socialization, relaxation, and general pleasure, all of which are key motivators for WTS, another important factor for WTS is the user perception that they are less harmful compared to cigarette smoking. One study showed that hookah is perceived by users to be the least addictive smoking product compared to e-cigarettes, marijuana, cigarettes, cigars, and smokeless tobacco products [12]. Another common notion is that the passing of tobacco smoke through water purifies the smoke before inhalation [13]. The low perceived risk of waterpipe tobacco smoking might be another important contributing factor to its usage, especially among adolescents [14]. However, one study has identified significantly higher yields of tar, nicotine, and carbon dioxide through WTS compared to cigarette smoking, which could lead to dependence, heart disease, lung disease, and cancer [15]. Another study in particular estimated the amount of carbon dioxide exhaled in one WTS session to be higher than that exhaled after smoking one pack of cigarettes [16]. Another key factor that influences the prevalence of WTS, especially in developing use habits for non-smokers, is the waterpipe flavor [17,18]. Among first-time waterpipe smokers in one study, 89% used a flavored product [18]. Another survey taken of waterpipe users revealed that roughly 80% of waterpipe decisions included flavor as a factor [19]. Among women using waterpipe/hookah products, 82% used waterpipe/hookah products sweetened with fruit flavors [20]. Flavored tobacco is especially effective in attracting younger users and poses a risk of exposing youth to other forms of tobacco use [18]. Additionally, one study on mice found that flavored waterpipe smoke was more toxic than unflavored waterpipe smoke and resulted in higher oxidative stress, which negatively alters lung function [21].

The available findings regarding the influence of flavor on waterpipe use patterns are similar to those on the influence of flavor on other tobacco product use patterns. For example, while 89% of first-time waterpipe smokers used a flavored product, 81% of first-time e-cigarette smokers used a flavored product, and 50% of first-time cigarette smokers used a flavored product [18]. Additionally, as flavored waterpipe smoke was found to be more toxic than unflavored waterpipe smoke for lung function, the use of flavored e-liquid in e-cigarettes was found to be more harmful for oral health than non-flavored e-liquid due to the presence of certain flavoring chemicals [22]. One study using data from the 2017 National Youth Tobacco Survey showed that flavored tobacco use was significantly correlated with greater risks of multiple tobacco product use [23]. Another study using the Tobacco Use Supplement to the Current Population Survey found that there were associations between tobacco flavor use and increased tobacco dependence [24].

Recognizing the appeal of sweeter, milder flavors that can mask the strong taste of tobacco, tobacco companies have used different flavors (such as fruits and candy) in tobacco products to attract the public (especially young people) to flavored tobacco smoking [25]. To specifically target the youth, young adults, and women, tobacco companies have developed different promotion strategies, for example, featuring celebrities and attractive models in their tobacco advertisements [26,27]. Due to the role of flavor in tobacco use initiation and tobacco dependence, as well as the negative health effects that it poses compared to non-flavored tobacco, certain measures have been implemented to restrict the sale of flavored tobacco. In 2009, the FDA implemented a national ban on flavored cigarettes besides menthol, which was found to reduce the probability of becoming a cigarette smoker by 17% and the probability of using any tobacco by 6% [28]. In 2020, to combat the rising popularity of electronic nicotine delivery systems, which was largely due to the appeal of flavored products to young people, the FDA passed a bill to restrict the sale of flavored electronic nicotine delivery systems (ENDSs) [29]. Although few studies have been conducted on the effects of this bill on ENDS use patterns, one study collecting data from six major US cities found an 8.4% decrease in e-cigarette use as a result of this bill [30]. Despite their growing popularity, and the similar influence of flavor on use patterns, tobacco dependence, and health effects, flavored waterpipe products have yet to be restricted by the FDA.

Given the rising popularity of WTS globally, the health risks associated with WTS, and its less stringent regulation compared to other similar tobacco products, it is important to examine the trends in waterpipe flavor prevalence, user opinions, and the general relevance of the product, as it can offer valuable insight to the nature of waterpipe use. Additionally, it is important to understand user opinions towards specific flavors, as this can help contextualize their prevalence on social media. As new findings are obtained regarding the usage of these products and as new flavors may emerge as well over time, it is important to monitor waterpipe smoking longitudinally to identify any drastic changes.

Social media is a reliable, accessible source for the mining of public opinions towards tobacco products. Twitter is one of the most popular social media platforms, with roughly 237 million active users worldwide as of July 2022 [31]. Twitter has been used in previous studies to understand user opinions of e-cigarettes and oral nicotine pouches [32,33]. Few studies have used Twitter to examine public opinion on waterpipe products, especially different waterpipe flavors [34]. In this study, by analyzing waterpipe-related tweets, we examined the prevalence of waterpipe-related tweets over time. Furthermore, we examined the prevalence of different waterpipe flavors mentioned on Twitter and their public perceptions. Our study contributes to the growing literature on waterpipe smoking to understand longitudinal trends in the prevalence and user perceptions of waterpipe smoking and the associated flavors on Twitter.

## 2. Materials and Methods

### 2.1. Data Collection

Waterpipe-related Twitter posts (tweets) were collected through the Twitter streaming application programming interface (API) using a list of keywords related to waterpipes, such as “waterpipe”, “shisha”, “narghile”, and “hookah” [34]. First, we extracted 1,394,016 waterpipe-related tweets from 9 March 2021 to 19 May 2022. Furthermore, we assigned each tweet to either a promotional or non-promotional category based on whether the tweet contained promotional words, such as “available now”, “grand opening”, “for sale”, etc., [32]. Altogether, we identified 1,158,884 non-promotional tweets and 235,132 promotional tweets (see the data-processing flowchart presented in Figure S1).

To investigate waterpipe flavors discussed on Twitter, we generated a list of flavors and brands by scraping the websites of different vendors [34]. Using flavors from this list as keywords, we extracted 21,891 tweets (17,746 non-promotional and 4145 promotional tweets) that mentioned at least one waterpipe flavor. Since some keywords generated noisy subsets, such as “tobacco” and “coffee”, we applied an additional filter for certain categories to remove noise, exclusively extracting tweets that matched flavor-related terminology, namely the term “flavor”. To better contextualize the flavors mentioned in the tweets, based on the previous well-established flavor wheel for e-cigarettes, we grouped each waterpipe flavor hierarchically into three levels [35]. Each specific flavor was categorized into a minor flavor category, each of which was grouped into an intermediate flavor category, each of which broadly fit into one major flavor category [32,34]. For example, a scraped flavor of “mango” would be grouped into the minor flavor category of “mango”, which is encompassed by the “tropical” intermediate flavor category, which is contained in the major flavor category of “fruit”. The nine major flavor categories were fruit, sweets, beverages, mint, alcohol, spices, tobacco, mixed, and others, based on Krüsemann et al.’s flavor wheel [35]. The mixed group included flavors that contained several flavor ingredients from other major categories, and the other group included flavors that could not be categorized into any of the previously mentioned groups.

### 2.2. Sentiment Analysis

As we were interested in user perceptions of waterpipe tobacco products, we only used non-promotional waterpipe-related tweets for further sentiment analysis. We conducted sentiment analysis on all non-promotional waterpipe-related tweets to better understand Twitter users’ attitudes towards general waterpipe smoking and specific waterpipe flavors. To investigate the attitudes towards general waterpipe smoking, we used VADER (the Valence-Aware Dictionary for Sentiment Reasoning), a lexicon-based sentiment analysis tool that is particularly well suited to social media data due to its ability to handle emoticons, slang, and emojis [36]. VADER sums over the polarity scores of every word in a user entry and outputs a sentiment score between −1 and 1, from most negative to most positive. Using this tool, we obtained a sentiment score for each non-promotional tweet and classified tweets in the range of −1.00 to −0.05 as negative, −0.05 to 0.05 as neutral, and +0.05 to 1.00 as positive. Before conducting sentiment analysis using VADER, data preprocessing was applied to all tweets to reduce the effect of extraneous information on sentiment analysis scores according to the recommendations from a previous study [37]. Specifically, numerical digits, hyperlinks of the form “www.link” and “https://link”, and tweet-specific syntax of the form “@username”, “RT: @username”, and “#hashtag” were removed from tweets using the twitter-preprocessor library. Additionally, HTML phrases such as “&amp” were translated to UTF-8 counterparts such as “&”.

Although general sentiment analysis is conducted at the sentence or document level, aspect-based sentiment analysis (ABSA) methodologies can assess sentiment towards specific entities within a string [38]. For example, observing the sentiment of the targets “mango” and “peach” within the context sentence “Most fruits are horrible, disgusting, and gross, but mango and peach are amazing”, the sentiments for the two target flavors should both be identified as positive, whereas the overall sentiment of the tweet could be considered negative. It is estimated that 40% of classification errors occur due to a lack of consideration of targets in sentiment classification [39]. Thus, we employed ABSA to contextualize user opinions toward specific waterpipe flavors, using 17,746 non-promotional waterpipe-related tweets that mentioned at least one waterpipe flavor. We used the Pytorch-ABSA tool due to its clear documentation and relatively better performance than other ABSA libraries. Rather than outputting a sentiment score, as VADER does, this library returns the overall sentiment of the input towards the target aspect (positive, negative, or neutral), along with the confidence of that classification.

## 3. Results

### 3.1. Temporal Analysis of Waterpipe-Related Tweets

**Figure 1:**
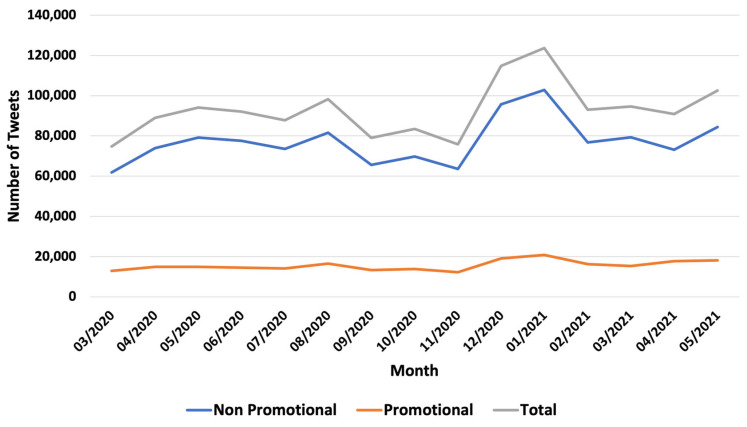
Number of tweets mentioning waterpipe over time.

Between March 2021 and May 2022, we identified 1,394,016 waterpipe-related tweets. Of these, we classified 235,132 tweets (17%) as promotional and 1,158,884 (83%) as non-promotional. As shown in Figure 1, the relative frequency of promotional posts and non-promotional posts appeared to be constant over time, with non-promotional posts being significantly more prevalent than promotional posts. Between March 2021 and May 2022, promotional and non-promotional waterpipe-related tweets increased steadily by an average of 3.8% and 3.7% per month, respectively. In addition, there was an obvious peak for both promotional and non-promotional tweets related to waterpipe smoking between December 2021 and January 2022.

### 3.2. Waterpipe Flavors Mentioned on Twitter

To examine the popularity of different waterpipe flavors mentioned on Twitter, we extracted tweets mentioning different waterpipe flavor categories in both non-promotional and promotional tweets. As shown in Table 1, among non-promotional posts, the fruit flavor was the most dominant major flavor group (6271/17,746, 34.18%), followed by sweets (3112/17,746, 17.86%), beverages (2671/17,746, 15.38%), mint (1740/17,746, 9.14%), mixed (1568/17,746, 8.64%), alcohol (1056/17,746, 5.57%), tobacco (544/17,746, 2.41%), and spices (411/17,746, 2.39%) respectively.

Similar to what was observed in the non-promotional tweets, fruit (1311/4145, 31.62%), sweets (815/4145, 19.66%), and beverages (657/4145, 15.85%) were the most mentioned flavor groups in the promotional tweets. The next most frequently mentioned flavor groups were tobacco (388/4145, 9.36%), mint (312/4145, 7.53%), mixed (255/4145, 6.15%), alcohol (202/4145, 4.87%), and spices (108/4145, 2.60%). Fruit, mint, and mixed flavors had significantly higher proportions in the non-promotional tweets compared to the promotional tweets (*p* value < 0.05). The sweets and tobacco flavors exhibited a significantly higher proportion in the promotional posts compared to the non-promotional posts (*p* value < 0.05).

**Figure 2:**
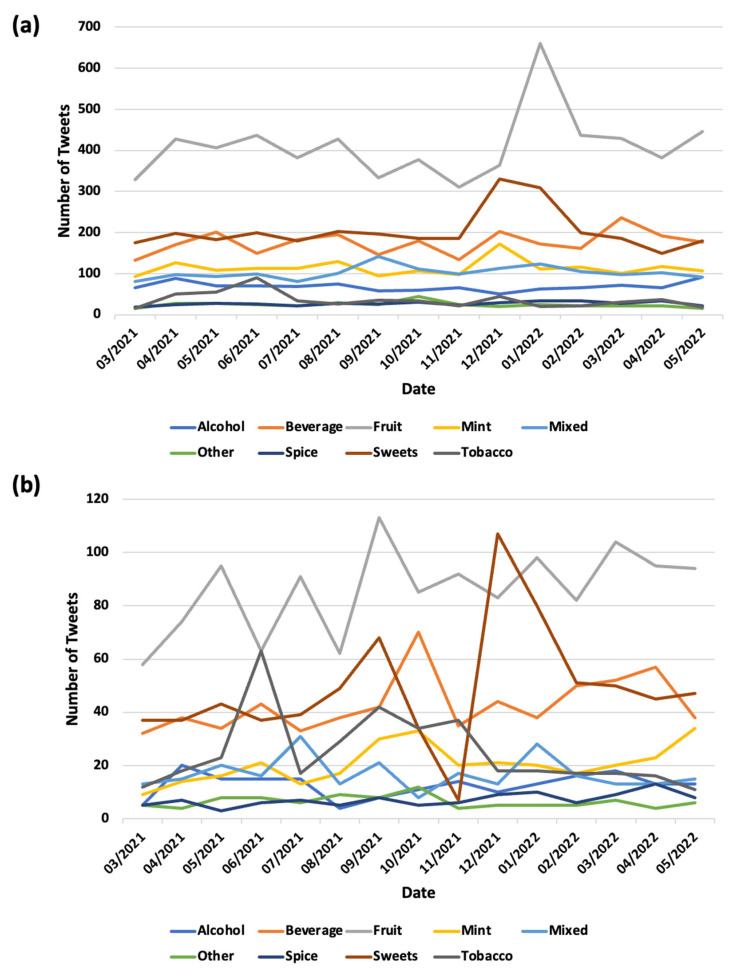
Longitudinal trends of waterpipe flavors mentioned on Twitter. (**a**) promotional tweets, (**b**) non-promotional tweets.

With the dynamic landscape of waterpipe use, we analyzed the mentions of different waterpipe flavors on Twitter longitudinally. As shown in Figure 2a, for promotional posts, all major flavor categories displayed somewhat random behavior in terms of monthly mentions. Although the fruit flavor generally remained dominant over time, there was an obvious peak for the fruit flavor around January 2022. As shown in Figure 2b, although the mentions of most flavors fluctuated among non-promotional tweets over time, the mentions of the fruit flavor exhibited a peak in September 2021, and the mentions of the sweet flavor were more prevalent from December 2021 to January 2022.

### 3.3. Sentiment Analysis of Waterpipe Flavors Mentioned on Twitter

Sentiment analysis was performed on the non-promotional tweets in order to better understand the user opinions towards WTS and specific flavor groups. Out of all non-promotional posts, we identified 39% of tweets (451,964/1,158,884) as neutral, 36% (417,198/1,158,884) as positive, and 23% (289,722/1,158,884) as negative. The proportion of positive tweets was significantly greater than the proportion of negative tweets in our dataset (*p* value < 0.05).

For flavor-containing tweets, the alcohol flavor category exhibited the highest percentage of positive tweets (74.62%), followed by mixed (72.13%) and fruit (65.62%) (Table 2). On the other hand, the tobacco flavor had the highest percentage of negative tweets (18.20%), followed by spices (15.82%) and mint (13.22%). When comparing the relative frequency of positive to negative tweets within each flavor group, the mixed flavor had the highest ratio of positive to negative tweets (7.11 to 1) and the tobacco flavor had the lowest ratio (2.43 to 1.00).

## 4. Discussion

By mining waterpipe-related tweets, we observed that waterpipe tobacco smoking was an increasingly popular topic on Twitter between March 2021 and May 2022, with a noticeable peak in January 2022. Among waterpipe-related tweets, we observed that roughly 17% were non-promotional tweets, and 83% were promotional tweets. By examining the frequency of waterpipe flavors mentioned, we showed that fruit, sweets, and beverages were the most mentioned flavor groups in promotional and non-promotional tweets, whereas tobacco and spices were the least mentioned flavor groups. Our temporal analysis showed that this trend remained constant over time, indicative of the consistent dominance of sweeter flavor profiles over unflavored or bitter ones in waterpipe-related discussions on Twitter. Sweet flavor profiles such as sweet and fruit displayed noticeably larger ratios of positive tweets to negative tweets than harsher, bitter flavor profiles such as tobacco and spice flavors.

Although social media has been used to assess longitudinal trends in the perception of other tobacco products such as e-cigarettes [32,40,41], few studies had investigated the longitudinal trends and public attitudes toward waterpipe flavors using social media data [34]. We showed that fruit, sweets, and beverages were the most mentioned waterpipe flavor groups on Twitter, which was similar to what was observed in a recent study on waterpipe-related discussions on Reddit and another study on flavored e-cigarettes on Twitter [32,34]. More importantly, we used aspect-based sentiment analysis (ABSA) rather than document-based sentiment analysis, which allowed for a more accurate contextualization of flavor mentions. As ABSA can target opinions towards specific entities in a document, an additional benefit of our approach was the ability to extract user sentiments towards multiple flavors in one document, as opposed to just one flavor. In contrast, traditional sentiment analysis methods used in other studies could only be applied to documents containing only one flavor in order to avoid ambiguity, since these methods are unable to handle situations where multiple flavors are mentioned in one tweet [32].

Our study showed that the mixed, alcohol, and fruit flavors had both the highest percentages of positive tweets and the highest ratios of positive to negative tweets, whereas tobacco and spices had the highest percentages of negative tweets and the lowest ratios of positive to negative tweets. That is, mixed, alcohol, and fruit flavors were the most preferred waterpipe flavors, whereas tobacco and spice flavors were the least preferred waterpipe flavors in our study. Although this finding shows general agreement with the existing literature on user preferences for hookah flavorings, namely, that more pleasant, sweeter flavor profiles are more favored by users over bitter ones [17,18,19,20], it differs slightly in terms of the order compared to Lu’s study, in which sentiment analysis was conducted on e-cigarette flavors [32]. For example, the most preferred flavors in our study were mixed, alcohol, and fruit, whereas the fruit flavor was the most preferred, followed by sweets and mint for e-cigarettes.

Interestingly, we noted that the most preferred flavors (mixed, alcohol, and fruit) had significantly lower proportions of mentions in promotional tweets than in the non-promotional tweets, whereas less preferred flavors (tobacco and sweets) had significantly higher proportions of mentions in promotional tweets than in non-promotional tweets. Although we could not find any specific feature in the dataset to explain this behavior, we speculate that this may have been due to the advertisement strategies employed by tobacco companies or online stores. Our logical speculation was that less preferred flavors are mentioned less among non-promotional tweets due to a lack of usage, and they are mentioned more frequently in promotional tweets to enhance sales due to their lack of popularity. On the other hand, more popular flavors are mentioned more in non-promotional posts due to their popularity among users, whereas they are advertised less due to the lack of necessity caused by consistent public demand.

As there may be specific factors that lead users to participate in waterpipe-related discussions on Twitter, and more importantly, the demographics of Twitter users are slightly different from the general population in the US, our results may be biased towards our sample, and the user opinions we observed in this study may not be generalizable to the entire population of waterpipe users. Additionally, as demographic information such as the age, race/ethnicity, and gender of Twitter users was not available through Twitter metadata, we were not able to investigate the prevalence of waterpipe flavor use among demographic groups, especially their public perceptions among different demographic groups, which might be important for future regulatory policy. In addition, as tweets are subject to a shorter character limit compared to other social media websites such as Reddit and other forums, our analysis of user opinions was limited to techniques that are conducive to short-form text such as sentiment analysis. In the future, other social media platforms such as Facebook and Reddit could be explored to better understand more specific topics of waterpipe-related discussion among social media users. Although we tried to include as many waterpipe flavors as possible in our waterpipe flavor list, some waterpipe flavors will be missed in our study since the flavors of waterpipe tobacco products are quickly evolving. In the future, it is necessary to have a complete waterpipe flavor list, and more importantly, to keep it updated. Finally, the prevalence of waterpipe flavors needs to be closely monitored longitudinally.

## 5. Conclusions

By identifying tweets mentioning waterpipe flavors and by conducting sentiment analysis, we were able to analyze the prevalence and user opinions towards waterpipe flavors mentioned on Twitter, providing a framework for how waterpipe flavor trends can be studied in the future. Given that flavor is a key motivator for waterpipe tobacco smoking, this framework can be extremely useful for public health officials and researchers to monitor the dynamic changes in waterpipe product marketing and user preferences. Although flavored waterpipe smoking has shown similar negative health effects, a similar influence on the initiation of waterpipe use, and a similar influence on tobacco dependency compared to cigarettes and electronic nicotine delivery systems, flavored waterpipe products are yet to be regulated by the FDA, whereas the sale of flavored cigarettes was banned by the FDA in 2009 and the sale of flavored e-cigarettes (other than tobacco and menthol flavors) was regulated by the FDA in 2020. Our findings displayed the generally positive sentiments of Twitter users towards flavored waterpipe products compared to non-flavored waterpipe products, which suggests that the regulation of flavored waterpipe tobacco products may be effective in decreasing their overall prevalence to protect public health, especially among youth. Considering the health risks posed by these waterpipe tobacco products, the potential for flavored products to attract youth, and the recent rise in the popularity of these products, this issue deserves much greater attention from public health authorities.

## Figures and Tables

**Table 1:** Waterpipe flavors mentioned on Twitter ^1^.

Major Flavor Group	Non-Promotional Posts, *n* (%)	Promotional Posts, *n* (%)
Fruit	6271 (34.18%)	1311 (31.62%)
Sweets	3112 (17.86%)	815 (19.66%)
Beverages	2671 (15.38%)	657 (15.85%)
Mint	1740 (9.14%)	312 (7.53%)
Mixed	1568 (8.64%)	255 (6.15%)
Alcohol	1056 (5.57%)	202 (4.87%)
Tobacco	544 (4.42%)	388 (9.36%)
Spices	411 (2.41%)	108 (2.60%)

^1^ The “Others” category was excluded.

**Table 2:** Sentiment analysis towards waterpipe flavors on Twitter ^1^.

Flavor Category	Tweet Count, *n*	Percent of Positive Tweets (%)	Percent of Negative Tweets (%)	Positive-to-Negative Ratio
Fruit	6271	65.62	12.03	5.45
Sweets	3112	60.41	12.18	4.96
Beverages	2671	65.21	12.65	5.15
Mint	1740	65.05	13.22	4.92
Mixed	1568	72.13	10.14	7.11
Alcohol	1056	74.62	12.50	5.97
Tobacco	544	44.30	18.20	2.43
Spices	373	61.56	15.82	3.89

^1^ The “Others” category was excluded.

## Data Availability

The social media data and Python code used for data analysis will be available upon reasonable request from the corresponding authors.

## Supplementary Materials

The following supporting information can be downloaded at: https://www.mdpi.com/article/10.3390/ijerph20075264/s1, Figure S1: Flowchart on Data Pre-Processing.
