# Supplementary figures and images for: Public Perceptions of Flavored Waterpipe Smoking on Twitter
Source: Int J Environ Res Public Health. 2023 Mar 27;20(7):5264. doi: 10.3390/ijerph20075264 (PMC10094574; doi:10.3390/ijerph20075264)

## Supplemental Figure

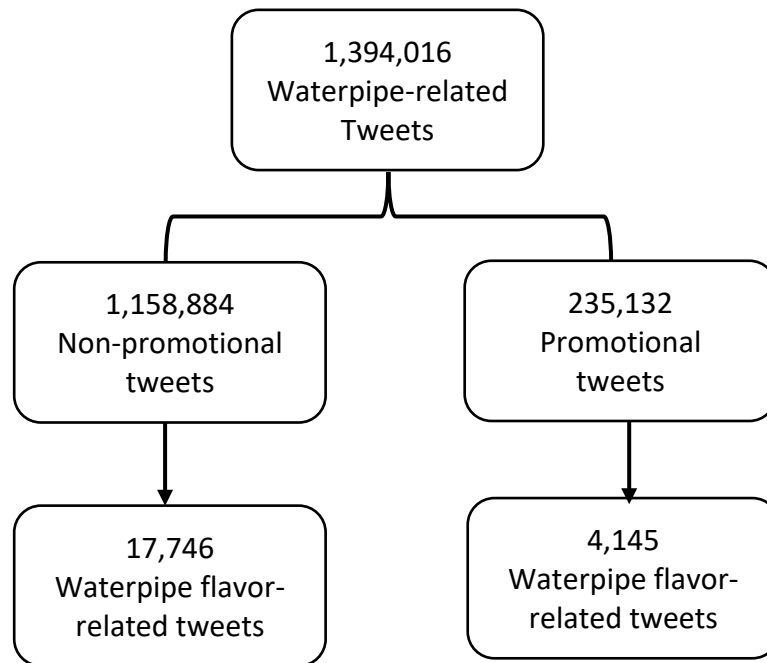

**Figure S1:** Flowchart on Data Pre-Processing.

Supplement: Supplementary file 1 [file ijerph-20-05264-s001.zip › ijerph-2231662-supplementary.pdf]
